# Heterotopic Ossification of the Elbow in a Patient With Cerebral Amyloid Angiopathy Following Intraparenchymal Hemorrhage: A Case Report

**DOI:** 10.7759/cureus.70643

**Published:** 2024-10-01

**Authors:** Stephen Howard, Ibrahim Chowdhury, Naomi Francois, Cecilia Ransom

**Affiliations:** 1 Physical Medicine and Rehabilitation, Lake Erie College of Osteopathic Medicine, Elmira, USA; 2 Rheumatology, Lake Erie College of Osteopathic Medicine, Elmira, USA; 3 Physical Medicine and Rehabilitation, Rochester Regional Health, Rochester, USA

**Keywords:** cerebral amyloid angiopathy, hemorragic stroke, heterotopic ossification (ho), intraparenchymal hemorrhage, precision radiation therapy

## Abstract

Heterotopic ossification (HO) is a rare complication that may be initially discovered in the acute inpatient rehabilitation setting, caused by the abnormal formation of bone tissue in non-skeletal areas of the body. It may be caused by trauma or a neurological injury. When treating a patient with a history of neurological disease complicated by an acute event, treatment should be tailored to suit the patient's needs and to avoid further harm. This case explores the complexity of HO in a patient diagnosed with cerebral amyloid angiopathy following an intraparenchymal hemorrhage (IPH) and highlights the option of management with radiation therapy when non-steroidal anti-inflammatory drugs (NSAIDs) are contraindicated, not only to treat but also to prevent the progression of HO in this patient.

## Introduction

Heterotopic ossification (HO) is a complication that can be seen in the acute inpatient rehabilitation setting [1]. The etiology of this complication includes burns, strokes, spinal cord injuries, traumatic brain injuries, amputation, and joint replacements [1]. There are multiple risk factors for the development of HO, including old age, the presence of spasticity, pressure ulcers, deep vein thrombosis, long bone fractures or prior injury to the area, edema, lack of mobility to the joint, and long-term coma [1]. HO has been shown to occur in approximately 53% of patients following a total hip arthroplasty, approximately 25% following spinal cord injury, and approximately 15% following traumatic brain injury [1]. Additionally, the likelihood of the development of HO is twice as great in females compared to males [1]. Common anatomical areas that are affected by HO include the hips, shoulders, and elbows [1].

The exact pathophysiology of HO is not fully understood but mainly stems from two common factors: trauma/surgery and neurological disorders [1]. Once the initial injury occurs, the pathophysiology can be explained: injury causes soft tissue damage and triggers an inflammatory response, including the release of bone morphogenic proteins, which leads to the proliferation and differentiation of mesenchymal cells into osteoblasts [2]. These osteoblasts cause bone formation in tissues, which then matures and remodels the muscle [2]. Imaging modalities such as X-rays, computerized tomography (CT) scans, and magnetic resonance imaging (MRI) are all useful for diagnosing HO. Patients should be assigned the most appropriate imaging modality based on their profile. A triple-phase bone scan is the most sensitive imaging modality for diagnosing patients with HO [1]. HO can be managed medically through non-steroidal anti-inflammatory drugs (NSAIDs) and bisphosphonates [1]. If the injury is beyond the scope of medication, surgery can be involved to remove the heterotopic bone. In patients who cannot tolerate NSAIDs, triple-phase radiation therapy is an appropriate option [1,2].

## Case presentation

A 74-year-old female presents to the Emergency Department following a right temporal intraparenchymal hemorrhage (IPH) two weeks prior to arrival, complicated by a history of cerebral amyloid angiopathy (CAA). Her past medical history includes chronic obstructive pulmonary disease, post-concussion syndrome, simple seizures, prior motor vehicle accidents, and chronic tobacco use.

**Figure 1:**
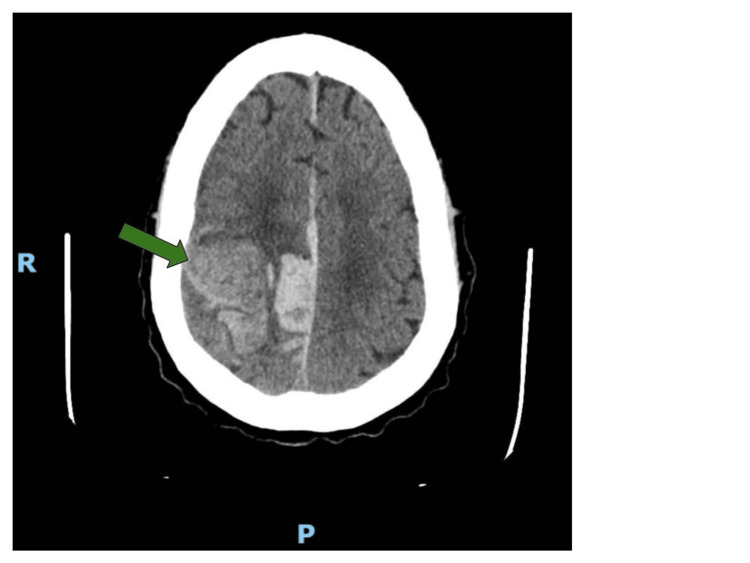
Computerized tomography of the head Significant acute right frontal and parietal intraparenchymal hematoma, with a considerable adjacent subfalcine subdural component measuring up to 20 millimeters (green arrow). There is a four-millimeter right to left midline shift.

She presented to the Emergency Department with a severe headache and acute onset of left-sided weakness. The symptoms began 25 minutes prior to her arrival at the emergency room via emergency medical services. She had previously been in a motor vehicle accident, and the MRI performed a week prior showed a subacute bleed with significant white matter changes. She was scheduled for outpatient neurology follow-up; however, these symptoms prompted her visit to the Emergency Department. Her vital signs showed a blood pressure of 202/110 mmHg, which was rechecked to be 199/121 mmHg. On physical examination, the patient was in no acute distress. Her right gaze was unable to cross the midline. Her pupils were equal and reactive. She was oriented to person and place but not to time. Her speech was dysarthric but appropriate. She had a large left facial droop. She had 0/5 strength in the left upper and lower extremities and 5/5 strength in the right upper and lower extremities. She also had diminished sensation on the left, appearing very neglectful. Her National Institutes of Health Stroke Score (NIHSS) was 12. She had not been taking any anticoagulants. A stroke alert was called, and the patient proceeded directly to a non-contrast head CT shown below (Figure 1).

The CT showed a large area of IPH with a 4 mm midline shift. Upon returning from the CT room, the patient's NIHSS worsened to about 20, with the addition of worsening facial droop, gaze deviation, and dysarthria. She was still maintaining her airway, with a SpO2 of 99% on room air. Neurology and neurosurgery were consulted. Neurology recommended a blood pressure goal of less than 140 mmHg systolic, with a 5 mg per hour dose of nicardipine. They also recommended a two-gram Keppra load, followed by 500 mg every 12 hours, given the patient’s epileptic seizure history. The neurosurgeon recommended no surgical intervention and admission to the surgical intensive care unit (ICU). 

The patient was admitted to the surgical ICU for monitoring, and her blood pressure was controlled with nicardipine. However, the patient had significant respiratory distress, with accessory muscle use requiring intubation and ventilation. She was followed by an ear, nose, and throat physician for tracheostomy placement to help facilitate weaning from the ventilator. A gastroenterology physician was also consulted for percutaneous endoscopic gastrostomy tube placement. The patient was hospitalized for a total of two months before admission to acute inpatient rehabilitation for comprehensive rehabilitation, consisting of physical therapy, speech therapy, occupational therapy, and neurophysiology evaluation.

**Figure 2:**
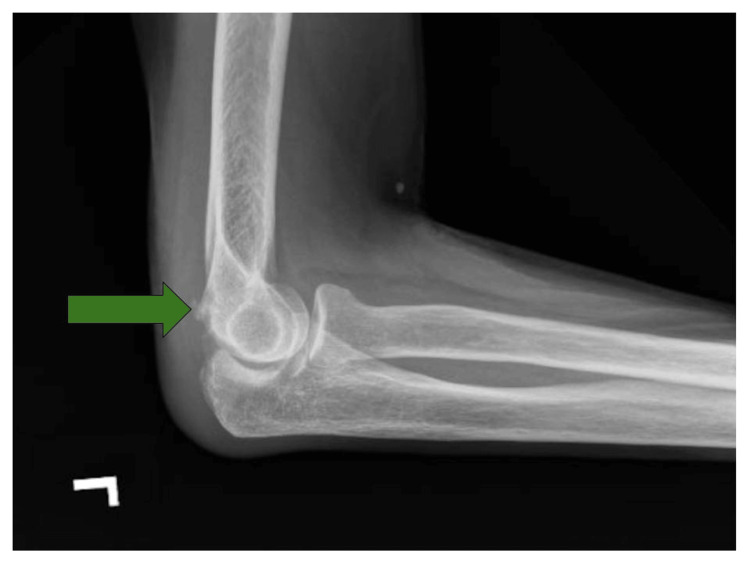
X-ray of the left elbow Slight spurring is noted along the dorsal aspect of the distal humerus (green arrow) which could be a sequela of prior injury or related to heterotopic ossification given history.

**Figure 3:**
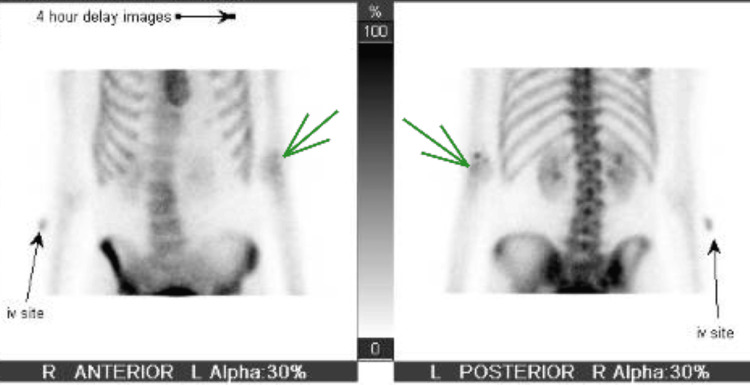
Triple phase bone scan Mild asymmetric increased uptake at the left elbow (green arrow) on delayed phase imaging, suggests heterotopic ossification.

During her stay in acute inpatient rehabilitation, the patient started to complain of pain in her left elbow. On physical examination, her left elbow range of motion was decreased. Flexion of her elbow to 90 degrees ended in a discrete endpoint with an increase in pain. HO was suspected, so an X-ray of her left elbow was ordered (Figure 2). The radiologist confirmed that this could indicate HO, so a triple-phase bone scan was ordered (Figure 3). The triple-phase bone scan confirmed HO. The patient’s history of CAA was a contraindication for NSAID use. Radiation oncology was consulted and recommended a single dose of radiation therapy to the left elbow.

The patient was treated with a single fraction of 7 Gy radiation to the left elbow joint area of HO, using a two-dimensional plane with 6 MV photons prescribed to the 100% isodose line. The patient was advised to follow up with outpatient orthopedics for a response assessment of HO. She tolerated the treatment well, without any immediate side effects.

## Discussion

Treatments such as indomethacin are ideal for treating patients with HO; however, the patient in the case described above was at significant risk of bleeding and hypertensive emergency due to her history of CAA [1]. Therefore, the risk of taking NSAIDs would outweigh the benefits, and radiation therapy was a more appropriate choice for treatment. It has been shown in multiple studies that radiation therapy can be used in the prevention of HO and can delay its progression [2]. The role of ionizing radiation in treating various benign diseases has been well-established and used for nearly a century [2]. Multiple studies have found that the use of a single dose of radiation therapy is the safest and most effective dosing for treating patients with HO [2]. However, it should be noted that radiation therapy can be toxic, as it can increase the risk of malignancy [2]. Therefore, for patients who are younger in age, radiation therapy should be carefully evaluated [2]. In the largest study analyzing a cohort of patients who received radiation therapy for HO prophylaxis, no in-field secondary malignancies were observed following treatment [3]. The researchers also concluded that the risk of malignancy following radiation therapy may not be as high as previously thought [3]. They suggest treating HO with radiation therapy in high-risk populations, as the risk of malignancy is not fully understood [3].

The patient described in this case had HO due to prolonged immobility from months of hospitalization and significant risk factors. The development of HO is a gradual process and can take months to show symptoms, as seen in this patient. For this reason, HO is more commonly seen in the acute rehabilitation setting as opposed to inpatient hospitalization. This patient's HO was appropriately treated with radiation therapy, as multiple clinical trials have shown that radiation therapy is more effective than NSAIDs in HO prevention [4]. The patients in this trial received 8 Gy in one fraction of radiation within 72 hours following acetabular surgery [4]. This single-dose treatment of radiation will prevent the overgrowth of bone and arrest the progression of HO and is recommended for patients who cannot take NSAIDs [4]. As this patient is indeed in a high-risk population for the progression of HO, and her inability to take NSAIDs makes her an ideal candidate for radiation therapy, it is important for physicians to recognize the gradual development process of HO and the use of radiation therapy in treating patients with HO who cannot take NSAIDs.

This patient suffered from an intracerebral hemorrhage likely secondary to her history of CAA. It has been shown that stable small vessel diseases, such as CAA, are the most common pathology underlying vascular cognitive impairments as a result of lacunar strokes or intracerebral hemorrhage [5]. CAA is an age-related small vessel disease that affects both the cerebral and leptomeningeal vessels due to the progressive deposition of amyloid-β [5]. It is imperative to accurately diagnose and treat CAA due to the risk of IPH. MRI is the gold standard imaging modality in patients who survive IPH with suspected CAA [5]. Despite the importance of treating patients with CAA, there are limited proven treatments [5]. Primary prevention of IPH includes hypertension control, which lowers the progression of white matter hyperintensities and significantly delays the onset of cognitive impairments secondary to this disease [5]. In the patient described above, she had a systolic blood pressure of greater than 200 mmHg upon presentation to the emergency room. Improvement in blood pressure control is crucial in patients with CAA. Thus, follow-up with a blood pressure cuff at home and follow-up with their primary care physician for blood pressure medication is essential.

Limitations of this article include the inability to follow up and analyze the range of motion of the patient's elbow flexion following radiation therapy. However, there is evidence that prophylactic radiation therapy is safe and more efficacious than NSAIDs in treating HO [4]. Strengths of this article include promoting the use of radiation therapy as a safe treatment for HO, as seen in the case presentation. Recent advances in the use of radiation therapy are scarce. Clinical trials, case reports, and other research modalities need to be conducted to advance the use of radiation therapy in treating patients with HO. 

## Conclusions

This patient, with a history of CAA, was appropriately treated for HO with the use of radiation therapy due to contraindications to NSAIDs. CAA often causes hypertensive crises and bleeding, which makes NSAIDs unsafe. Therefore, radiation therapy is the most optimal treatment choice for this patient. The benefits far outweigh the risks when it comes to using radiation therapy in high-risk patients to treat HO. This case promotes the importance of treating every case of HO on an individual basis and tailoring the treatment plan based on the specific patient. With this case report, we hope to inform physicians of the use of radiation therapy in treating patients with HO and to raise awareness of when it is indicated to treat patients with radiation therapy.
